# Time-Dependent Changes of Laboratory Parameters as Independent Predictors of All-Cause Mortality in COVID-19 Patients

**DOI:** 10.3390/biology11040580

**Published:** 2022-04-11

**Authors:** Nathaly Limon-de la Rosa, Eduardo Cervantes-Alvarez, Osvely Méndez-Guerrero, Miguel A. Gutierrez-Gallardo, David Kershenobich, Nalu Navarro-Alvarez

**Affiliations:** 1 Department of Gastroenterology, Instituto Nacional de Ciencias Médicas y Nutrición Salvador Zubirán, Mexico City 14080, Mexico; monny.limon@comunidad.unam.mx (N.L.-d.l.R.); eduardo.c@comunidad.unam.mx (E.C.-A.); ines.mendezg@incmnsz.mx (O.M.-G.); david.kershenobichs@incmnsz.mx (D.K.); 2 PECEM, Facultad de Medicina, Universidad Nacional Autónoma de México, Mexico City 04360, Mexico; bigbang.magg@gmail.com; 3 School of Medicine, Universidad Panamericana, Campus México, Mexico City 03920, Mexico; 4 Department of Surgery, University of Colorado Anschutz Medical Campus, Denver, CO 80045, USA

**Keywords:** COVID-19, biomarkers, clinical course, C reactive protein, inflammation markers

## Abstract

**Simple Summary:**

Several independent predictors of mortality for COVID-19 patients have been identified. However, those markers are usually parameters evaluated upon hospital admission, and clinical and biochemical parameters during the clinical course of the patients are usually neglected. To know the whole picture of COVID-19 patients it is important to evaluate the clinical course, as this will dictate how the patient progresses. We identify herein clinical and laboratory parameters from admission to discharge, or death, that distinguish between survivors and non-survivors of COVID-19, including those with independent ability to predict mortality.

**Abstract:**

Independent predictors of mortality for COVID-19 patients have been identified upon hospital admission; however, how they behave after hospitalization remains unknown. The aim of this study is to identify clinical and laboratory parameters from admission to discharge or death that distinguish survivors and non-survivors of COVID-19, including those with independent ability to predict mortality. In a cohort of 266 adult patients, clinical and laboratory data were analyzed from admission and throughout hospital stay until discharge or death. Upon admission, non-survivors had significantly increased C reactive protein (CRP), neutrophil count, neutrophil to lymphocyte ratio (NLR) (*p* < 0.0001, each), ferritin (*p* < 0.001), and AST (aspartate transaminase) (*p* = 0.009) compared to survivors. During the hospital stay, deceased patients maintained elevated CRP (21.7 mg/dL [admission] vs. 19.3 [hospitalization], *p* = 0.060), ferritin, neutrophil count and NLR. Conversely, survivors showed significant reductions in CRP (15.8 mg/dL [admission] vs. 9.3 [hospitalization], *p* < 0.0001], ferritin, neutrophil count and NLR during hospital stay. Upon admission, elevated CRP, ferritin, and diabetes were independent predictors of mortality, as were persistently high CRP, neutrophilia, and the requirement of invasive mechanical ventilation during hospital stay. Inflammatory and clinical parameters distinguishing survivors from non-survivors upon admission changed significantly during hospital stay. These markers warrant close evaluation to monitor and predict patients’ outcome once hospitalized.

## 1. Introduction

The current outbreak of coronavirus disease (COVID-19), reported first in Wuhan, China in December 2019, has been identified as an international emergency because of its high infection and mortality rate [1]. The risk of severe illness increases with age and the presence of underlying comorbidities [2,3]. Consequently, the public health system has been overloaded due to the high prevalence of diabetes, obesity, and chronic diseases worldwide [4,5,6].

Clinical manifestations among severe acute respiratory syndrome-2 (SARS-CoV-2)-infected patients range from asymptomatic infection to acute respiratory distress syndrome (ARDS), and even death [7,8]. Identifying potential biochemical indicators leading to disease progression or death based on routine laboratory tests in COVID-19 hospitalized patients has been of great interest due to low cost and availability. Elevations of inflammation-related proteins, such as C-reactive protein (CRP), ferritin and D-dimer [9,10,11,12], together with neutrophilia and lymphopenia [13,14] have been shown to correlate with disease severity [14]. These markers have been validated using laboratory data acquired only upon hospital admission without considering changes in laboratory data during hospitalization.

In this study we sought to evaluate the prognostic utility of clinical and laboratory characteristics both on admission and during a patient’s hospital stay to obtain the whole clinical picture of the course of the disease and to assess and predict patients’ outcome more accurately. The overall goal was to identify useful prognostic factors that could allow us to efficiently allocate resources to decrease mortality in COVID-19 patients.

## 2. Materials and Methods

### 2.1. Study Design and Groups

Patients >18 years admitted between April and July 2020 at Instituto Nacional de Ciencias Médicas y Nutrición Salvador Zubirán (INCMNSZ) in Mexico were included. Eligibility criteria were as follows: (a) laboratory-confirmed SARS-CoV-2 by real-time PCR; (b) computerized tomography (CT) and clinical characteristics compatible with COVID-19; and (c) definitive clinical outcome including death or discharge. Patients were divided into two groups: survivors and non-survivors (Supplementary Figure S1). This study was approved by INCMNSZ’s Research Ethics Committee (No. GAS-3385-20-20-1) and complied with the provisions of the Declaration of Helsinki.

### 2.2. Data Collection

Patients’ demographic (age, gender, comorbidities), clinical (treatment for COVID-19 prior to hospital admission, length of stay at hospital, intensive care unit (ICU) admission, use of vasopressors, requirement of invasive mechanical ventilation and enteral nutrition), radiological (chest CT findings) and laboratory and outcome data (death or hospital discharge) were collected from electronic medical records. All laboratory data were requested by the attending physician and samples were sent to the hospital’s central laboratory. This included the following tests: arterial blood gas, complete blood count, liver (aspartate transaminase [AST], alanine aminotransferase [ALT], alkaline phosphatase [ALP] and total bilirubin) and inflammation-related parameters (CRP and ferritin). All information was recorded in a specific database and was reviewed by 2 independent investigators to verify the correct collection of the data.

### 2.3. Outcome Measures and Operational Definitions

The primary outcome was all-cause mortality during hospitalization. Clinical and laboratory data were analyzed at two time-points: on admission and during hospitalization. For each variable analyzed, the measurement on admission was defined as the mean of all measurements, which occurred within the first 48 h after admission. The measurement of the variable labeled hospital stay was defined as that obtained from the mean of all measurements that were performed from day 2 to 48 h, prior to discharge or death (Supplementary Figure S1).

### 2.4. Statistical Analysis

Continuous variables are reported according to data distribution (mean ± standard deviation (SD) or median and interquartile range [IQR]). Categorical values are reported as *n* (%). Asymmetry values, kurtosis, and Kolmogorov–Smirnov test were used to analyze variable distribution. Tests of significance include Pearson’s Chi-squared test for categorical variables and Student’s test or Mann–Whitney U test for continuous variables, depending on data distribution. The two analyzed time-points (admission and hospital stay) were compared with the Wilcoxon signed-rank test. A smoothing spline model was generated with four knots between mortality and inflammatory parameters; if non-linear behavior, variables were entered as dichotomic with the appropriate cut-off value. Bivariate analyses were used and variables with *p* < 0.20 were included in multivariate logistic regression analysis to determine independent predictors; goodness of the fit was tested with the Hosmer–Lemeshow test. From these analyses, continuous variables known to independently predict the patient’s outcome were further analyzed with receiver operating characteristic (ROC) curves to determine their discriminating power, and cut-off values were established that correspond to the value with the highest Youden index, which maximizes sensitivity and specificity. Analyses were performed with SPSS (version 24, SPSS Inc., Chicago, IL, USA) and GraphPad Prism (version 8, GraphPad Software, California, CA, USA). A *p* value < 0.05 was determined as statistically significant.

## 3. Results

### 3.1. Demographic and Clinical Characteristics

All 266 patients with confirmed COVID-19 included in the study had pneumonia and abnormal CT findings, mainly identified by a ground glass opacity pattern upon hospital admission. Demographic and clinical characteristics are presented in (Table 1). The mean age was 53 ± 13 years, with 66% male. The median body mass index was 30 reflected in an elevated percentage of obesity (54%). The primary outcome for the 266 patients was as follows: 93 (35%) died 9 days after admission (non-survivor group) [IQR 5–17] and 173 (65%) survived and were discharged at a median of 12 days [IQR 7–25] after admission. The most common comorbidities were hypertension, diabetes, and hepatic disease, with diabetes being more common in deceased patients (37% vs. 23%, *p* = 0.023). As non-survivors presented a more severe disease stage, their therapeutic treatment frequently included vasopressors (*p* < 0.0001), invasive mechanical ventilation (*p* < 0.0001) and enteral nutrition (*p* < 0.0001) when compared to survivors. An interesting observation was that survivors received antivirals in a higher proportion than non-survivors (*p* = 0.026) prior to hospital admission, with oseltamivir showing statistical significance (0.038) among groups (Supplementary Table S1).

### 3.2. Abnormal Laboratory Findings upon Admission Related to a Higher Mortality Risk

**Figure 1:**
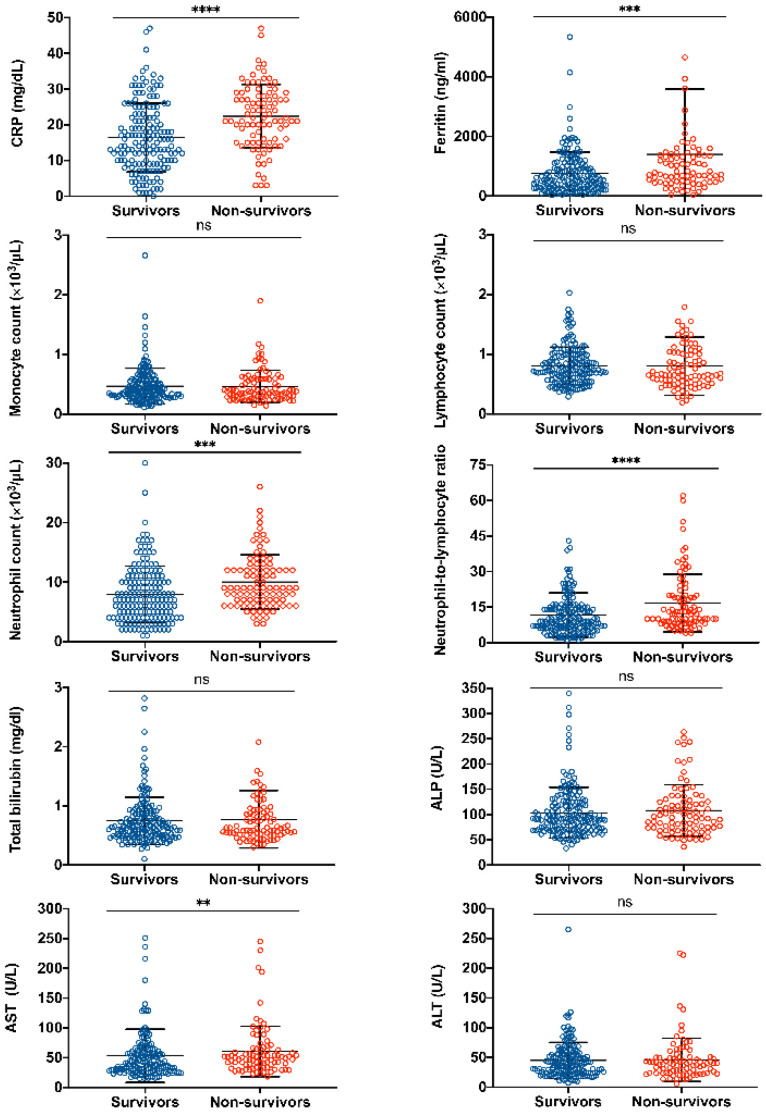
Laboratory parameters of COVID-19 patients evaluated upon hospital admission. C-reactive protein, CRP; alkaline phosphatase, ALP; aspartate aminotransferase, AST; alanine aminotransferase, ALT. Data are presented as median with IQR, two-tailed Mann–Whitney U test was performed. **** *p*< 0.0001, *** *p*< 0.001, ** *p*< 0.01.

Upon admission, increased levels of inflammation-related parameters such as CRP and ferritin were significantly higher in the non-survivor group when compared with survivors (*p* < 0.0001 and *p* < 0.001, respectively). In addition, non-survivors had a higher neutrophil count (*p* < 0.0001), an elevated NLR (*p* < 0.0001) and aspartate transaminase (AST) (*p* = 0.009) compared to survivors. No statistical difference was observed in the levels of lymphocyte and monocyte count, total bilirubin, alanine aminotransferase (ALT) or alkaline phosphatase (ALP) among groups (shown in Figure 1).

### 3.3. Abnormal Laboratory Findings during Hospital Stay Related to a Higher Mortality Risk

**Figure 2:**
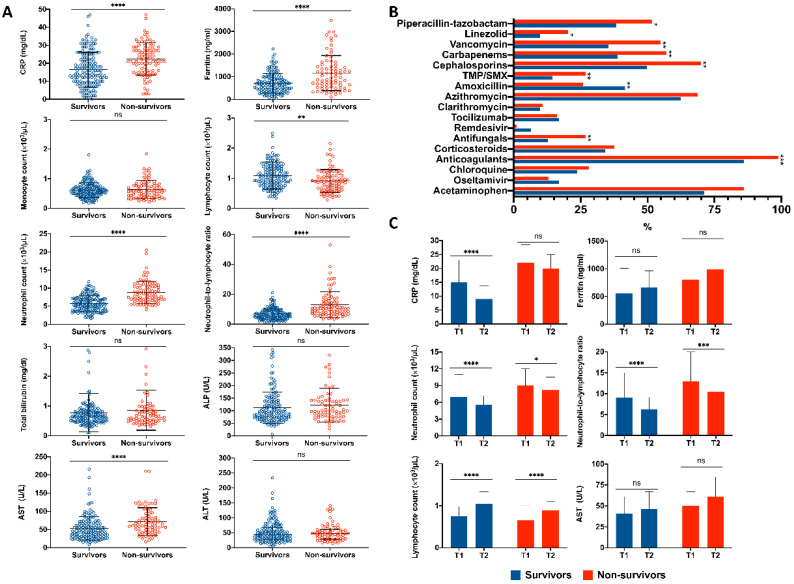
Laboratory parameters and treatment of COVID-19 patients evaluated during hospitalization. (**A**) Serum levels of laboratory parameters and its distribution in survivors and non-survivors. Data are presented as median with IQR, two-tailed Mann–Whitney U test was performed (**B**) Therapeutic treatment among COVID-19 patients and its frequency between survivors and non-survivors. (**C**) Time-dependent changes in CRP, Ferritin, neutrophil count, Neutrophil-to-lymphocyte ratio, lymphocytes and AST between survivors and non-survivors. T1: admission data and T2: hospitalization. Wilcoxon-signed rank test was performed. C-reactive protein, CRP; alkaline phosphatase, ALP; aspartate aminotransferase, AST; alanine aminotransferase, ALT. **** *p*< 0.0001, *** *p*< 0.001, ** *p*< 0.01.

To evaluate the severity of the patient’s clinical course, measurements of these variables during their hospital stay were further analyzed. Similar to data upon admission, we found significantly higher levels of the inflammation-related parameters CRP and ferritin (*p* < 0.0001 each), as well as a greater neutrophil count (*p* < 0.0001), a decreased lymphocyte count (*p* = 0.0014), and an elevated NLR (*p* < 0.0001) in non-survivors compared to survivors. Additionally, non-survivors had more frequent elevations in AST than survivors (*p* < 0.0001) (shown in Figure 2A).

To assess the clinical status of these patients, treatment during hospitalization was analyzed between survivors and non-survivors. Deceased patients received acetaminophen (*p* = 0.006) and broad-spectrum antibiotics more frequently, which included cephalosporins (*p* = 0.002), carbapenems (*p* = 0.005), vancomycin (*p* = 0.003), piperacillin-tazobactam (*p* = 0.038), trimethoprim-sulfamethoxazole (TMP/SMX) (*p* = 0.015), and linezolid (*p* = 0.023), as well as antifungals (*p* = 0.005), and anticoagulants (*p* = 0.001). Conversely, survivors received amoxicillin in a significantly higher proportion (*p* = 0.011). No statistical difference for antivirals or repurposed drugs (remdesivir and tocilizumab, respectively) was found between groups (shown in Figure 2B).

We next evaluated changes in laboratory data over time and compared differences between groups to determine if these variations could identify those in a more critical state or prone to progress. Compared to data upon admission, CRP was significantly lower throughout hospitalization (15.8 mg/dL [IQR 9.7–23.2] vs. 9.3 [IQR 5.5–13.7]) in those who survived (*p* < 0.0001). On the other hand, CRP remained elevated (21.7 mg/dL [IQR 15.1–28.3] vs. 19.3 [13.9–24.9]) from day 1 and throughout hospital stay, as no significant differences were observed between the two time points (*p* = 0.060) in non-survivors. Changes in neutrophil count for both survivors and non survivors were significant, with lower values found during hospital stay compared to admission data (*p* < 0.0001 and *p* = 0.024, respectively). However, non-survivors had significantly higher values at both time points when compared to survivors. The same was true for NLR (*p* < 0.0001 and *p* = 0.001 in non-survivors). The lymphocyte count increased from admission to hospital stay in both groups (*p* < 0.0001), being more pronounced in survivors. No statistical differences between groups were observed when analyzing AST and ferritin (shown in Figure 2C).

### 3.4. Clinical and Laboratory Predictors Associated with Mortality Due to COVID-19

**Figure 3:**
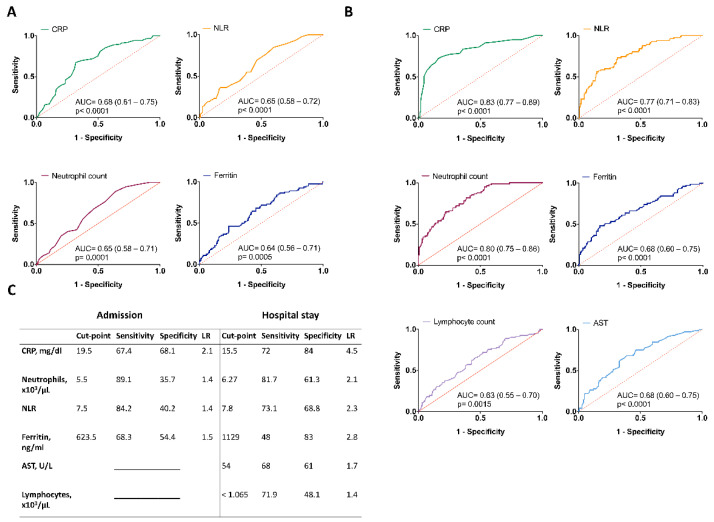
ROC curves of laboratory markers for COVID-19 mortality. (**A**) Data obtained upon hospital admission. (**B**) Data obtained upon hospitalization. (**C**) Summary of cut-off values, sensibility, specificity, and likelihood ratio.

To assess the predictive ability of CRP upon admission for in-hospital mortality, a ROC curve was plotted. The AUC was 0.68 (95% CI 0.61–0.75, *p* < 0.0001), with a cut-off value of 19.5 mg/dL with 67.4% sensitivity, 68.1% specificity and 2.1 LR. Likewise, we found that the neutrophil count (AUC = 0.65, *p* = 0.0001), NLR (AUC = 0.65, *p* < 0.0001) and ferritin (AUC = 0.64, *p* = 0.005) levels upon admission had discriminative power to predict mortality (shown in Figure 3A). Furthermore, values of different clinical and laboratory parameters evaluated during the hospital stay were plotted in ROC curves (shown in Figure 3B). Among those parameters with discriminative power, we found neutrophil count (*p* < 0.0001), NLR (*p* < 0.0001), ferritin (*p* < 0.0001), AST (*p* < 0.0001), lymphocyte count (*p* = 0.0015) and CRP (*p* < 0.0001), the latter having the highest AUC 0.83 (95% CI 0.77–0.89, *p* < 0.0001), and a cut-off value of 15.5 mg/dL with 72.1% sensitivity, 83.9% specificity and a LR of 4.5. The chosen cut-offs for the rest of the parameters are presented in Figure 3C.

Independent predictors of mortality obtained with data upon hospital admission and hospitalization were determined after multivariable logistic regression analysis. Given that the smoothing spline of CRP showed a non-linear relationship with mortality, this variable was entered as binary according to its cut-off value. The analysis of clinical and laboratory parameters upon admission revealed that patients with diabetes, CRP above 19.5 mg/dL and elevated ferritin levels were independently associated with mortality secondary to COVID-19 (*p* = 0.013, *p* < 0.0001 and *p* = 0.029, respectively). Accordingly, hospitalization data showed that CRP values above 15.5 mg/dL (*p* < 0.0001), a high neutrophil count (*p* < 0.0001), the requirement of invasive mechanical ventilation (*p* < 0.0001) and a longer hospital stay (*p* < 0.0001) are independent predictors of in-hospital mortality (Supplementary Table S2).

## 4. Discussion

In this study we evaluated the changes in laboratory data and clinical characteristics of 266 COVID-19 patients from admission to discharge or death. As the clinical status of the patient may vary significantly during hospitalization, we found important differences in several clinical and laboratory values between survivors and those who died during their hospital stay. On admission, elevated levels of CRP, AST, NLR, hyperferritinemia and neutrophilia were significantly related to in-hospital death. Data obtained during the patients’ hospital stay was consistent with those findings upon admission and revealed that lymphocytopenia, a higher frequency of interventions (vasopressors, enteral nutrition, and invasive mechanical ventilation), and broad-spectrum antibiotics were more common in non-survivors. A significant difference was observed in the hospital stay length, as non-survivors had a shorter stay. The latter indicates that patients arrived in a more critical condition and died soon after hospitalization.

Previous studies have identified CRP levels upon admission as an independent marker of disease progression [15], adverse outcomes and mortality [12,16], or as a marker to discriminate between severe and mild disease [17]. In this study we confirmed that elevated CRP values correlate with disease progression and poor prognosis with a cut-off value of 19.5 mg/dL upon hospital admission. However, we also found that maintaining elevated CRP values (>15.5 mg/dL) during hospitalization was an independent predictor of mortality. After observing such elevated CRP values and knowing that bacterial infections could be contributing to this event [18], we analyzed the frequency of secondary infections among groups during hospitalization. Both groups presented a similar infection rate (23.1 vs. 22.5%) with no significant difference among groups (*p* = 0.999), thus indicating that CRP values are unrelated to superinfection, accurately indicating COVID-19 progression.

According to these observations and the fact that both measurements of CRP (admission and during hospitalization) showed a non-linear relationship with mortality, as visualized on their smoothing splines, an optimal cut-off may be difficult to establish, which may explain why many authors have reported different values. Nonetheless, CRP values are more stable and useful for predicting mortality if measured during hospitalization. The utility of this inflammatory marker in COVID-19 is further apparent in the significant reduction observed in those who survived when the values upon admission are compared with multiple measurements during hospitalization.

Ferritin has been demonstrated to be unreliable in predicting fatal outcomes [19]; in our cohort, its values on both analyzed time-points had a significant AUC but it was not significantly different among groups. Conversely, neutrophilia and lymphocytopenia represent important markers to be considered during the natural history of the disease, the latter correlating with disease progression and death [9,20]. Previous studies have identified neutrophilia upon admission as an independent predictor of mortality in COVID-19 patients [21]. This phenomenon is common in viral infections, in which neutrophil-attracting chemokines increase to promote neutrophil infiltration into affected tissue as a response to the pathogen [22]; this being one of the main reasons the neutrophil count also correlates with the severity of the disease [17]. In this study, neutrophil count between survivors and non-survivors was significantly different. However, only an elevated neutrophil count during the hospital stay was an independent predictor of mortality in COVID-19 patients, highlighting the fact that a sustained inflammatory response is detrimental to the patient’s clinical course. NLR has been used as an indicator of systemic inflammation and severe COVID-19 [20,23,24]. Accordingly, higher NLR values at both timepoints were associated with an increased mortality risk.

Antivirals were more commonly prescribed to those with a better clinical outcome, as only 5% of non-survivors received oseltamivir, acyclovir, or amantadine. Evidence has shown that when prescribed during hospitalization, oseltamivir does not improve the patients’ disease severity [25], which was verified in our cohort, as its use did not show any amelioration of symptoms or clinical improvement.

We found that diabetes is an independent predictor of mortality. A known fact in COVID-19 patients is that their disease course may vary significantly, not only by the degree of activation of their immune system, but also by comorbidities that add to the system’s inflammatory response [26,27]. Indeed, COVID-19 patients with diabetes, a disease associated with metabolic syndrome, have higher mortality. This could be explained by the presence of higher levels of inflammatory cells and markers [28]. Diabetes and hypertension are also considered risk factors for COVID-19, which may be explained by an upregulation of the functional receptor ACE2 used by SARS-CoV-2 [29]. Due to the worldwide epidemic of obesity, immediate therapeutic measures and a closer monitoring of metabolic patients may be especially relevant to prevent progression of COVID-19 and death.

An emerging method of statistical analysis is machine learning and the use of artificial intelligence, which have consistently stood out as innovative means of clinical course prediction with the advent of the COVID-19 pandemic [30]. Their application is directed towards the processing of big data which facilitates the medical decision-making process, thus being convenient, especially now, when the need to efficiently assign resources is of utmost importance. However, their interpretability and accessibility among clinicians is still limited. This study highlights the relationship of several conventional laboratory and clinical parameters with mortality due to COVID-19, both on admission and during the patients’ hospital stay, thus providing a clinician-friendly solution that can help guide the patient-care rationale more readily. As with machine learning, these findings are only meant to serve as a support and the ultimate decision should be met according to a comprehensive and individualized interpretation of the patient’s clinical course. Interestingly, concordance is found between the independent predictors of mortality herein reported and those variables reported as having the highest association according to machine-learning algorithms, including CRP levels [31,32] and ferritin [33].

As a main limitation of this study, the findings described derive from a moderate-sized cohort that originates from a single center and must be further validated in a larger group of patients. Nonetheless, the significance observed in these analyses suggest relevant differences in clinical markers between COVID-19 non-survivors and survivors, which are also evident when measurements upon admission and during the hospital stay are contrasted, hence proving their usefulness in the assessment of a patient’s outcome.

## 5. Conclusions

In this cohort study, we point out the importance of evaluating patients throughout the entire clinical course, as the laboratory parameters evaluated showed significant changes during the patients’ hospitalization. Among the clinical and laboratory parameters evaluated, we report that elevated CRP values, neutrophil count, hyperferritinemia, diabetes, requirement of invasive mechanical ventilation and the length of hospitalization contribute to in-hospital death. The identification and continuous evaluation of such parameters may help clinicians to better allocate resources and avoid progression to a poor prognosis in COVID-19 patients.

## Figures and Tables

**Table 1:** Clinical characteristics and outcomes of COVID-19 patients.

	Total (*n* = 266)	Survivors (*n* = 173)	Non-Survivors (*n* = 93)	*p* Value
**Age (yr.)—mean (SD)**	53 (13)	52 (13)	55 (13)	0.099
**Sex, male—No. (%)**	175 (66)	106 (61)	69 (74)	**0.034**
**Weight (kg)—median (IQR)**	82 (73–92)	82 (73–;90)	84 (70–95)	0.256
**Smoking history—No. (%)**				
**Current**	29 (11)	18 (10)	11 (12)	0.477
**Former**	60 (23)	39 (23)	21 (23)	0.459
**Body mass index (kg/m^2^)—median (IQR)**	30 (27–34)	30 (28–33)	31 (27–34)	0.424
Normal (%)	34 (13)	24 (14)	10 (11)	0.786
Overweight (%)	88 (33)	57 (33)	31 (33)	
Obesity (%)	144 (54)	92 (53)	52 (56)	
**Days from symptom onset to admission—median (IQR)**	8 (5–13)	8 (5–14)	7 (5–11)	0.504
**Days from admission to discharge—median (IQR)**	10 (6–23)	12 (7–25)	9 (5–17)	**0.004**
**Pre-existing diseases—No. (%)**				
Diabetes	74 (28)	40 (23)	34 (37)	**0.023**
Hypertension	87 (33)	57 (33)	30 (32)	0.892
Hepatic disease	12 (5)	7 (4)	5 (5)	0.758
Alcoholism	23 (9)	13 (8)	10 (11)	0.239
Asthma	4 (2)	3 (2)	1 (1)	0.999
Cancer	8 (3)	5 (3)	3 (3)	0.999
**Symptomatic treatment prior admission—No. (%)**				
Nonsteroidal anti-inflammatory agents and acetaminophen	145 (55)	101 (58)	44 (47)	0.094
Antibiotics	173 (65)	113 (65)	60 (65)	0.999
Antihistamines	19 (7)	14 (8)	5 (5)	0.466
Antivirals	31 (12)	26 (15)	5 (5)	**0.026**
Antitussives	29 (11)	22 (13)	7 (8)	0.222
Antiasthmatics	30 (11)	21 (12)	9 (10)	0.685
Chloroquine	8 (3)	8 (5)	0 (0)	0.054
Corticosteroids	51 (19)	34 (20)	17 (18)	0.871
Others	75 (28)	48 (28)	27 (29)	0.887
**Interventions—No. (%)**				
Invasive mechanical ventilation	151 (57)	75 (43)	76 (82)	**<0.0001**
Use of vasopressors	147 (55)	75 (43)	72 (77)	**<0.0001**
Enteral nutrition	136 (51)	72 (42)	64 (69)	**<0.0001**

Bold values represent *p* < 0.05.

## Data Availability

All data generated or analyzed during this study are included in this article [and/or] its supplementary material files.

## Supplementary Materials

The following supporting information can be downloaded at: https://www.mdpi.com/article/10.3390/biology11040580/s1, Table S1: Antiviral prescription prior hospital admission; Table S2: Logistic regression for clinical factors on admission and during the hospital stay; Figure S1: Flow chart.
