# Supplementary material for: Time-Dependent Changes of Laboratory Parameters as Independent Predictors of All-Cause Mortality in COVID-19 Patients
Source: Biology (Basel). 2022 Apr 11;11(4):580. doi: 10.3390/biology11040580 (PMC9028239; doi:10.3390/biology11040580)
Supplement: Supplementary file 1 [file biology-11-00580-s001.zip › biology-1647114-supplementary.pdf]

Supplementary Table S1. Antiviral prescription prior hospital admission

	Total (n= 266)	Survivors (n= 173)	Non-survivors (n= 93)	*p* value
**Oseltamivir – no. (%)**	17 (6)	15 (9)	2 (2)	**0.038**
**Amantadine – no. (%)**	7 (3)	6 (4)	1 (1)	0.427
**Acyclovir – no. (%)**	6 (2)	5 (3)	1 (1)	0.668
**Ribavirin – no. (%)**	3 (1)	2 (1)	1 (1)	0.999

Bold values represent $p<0.05$.

Supplementary Table S2. Logistic regression for clinical factors on admission and during the hospital stay

	Admission						Hospital stay					
	Bivariate			Multivariable			Bivariate			Multivariable		
**Variable**	**OR**	**95% CI**	**p value**	**aOR**	**95% CI**	**p value**	**OR**	**95% CI**	**p value**	**aOR**	**95% CI**	**p value**
**Age, mean**	1.02	0.997 – 1.036	0.104									
**Sex, male**	1.82	1.042 – 3.169	**0.035**									
**Diabetes**	1.90	1.096 – 3.298	**0.022**	2.31	1.191 – 4.462	**0.013**						
**CRP (binary)**	4.42	2.542 – 7.672	**<0.0001**	4.93	2.684 – 9.036	**<0.0001**	13.57	7.074 – 26.056	**<0.0001**	8.35	3.374 – 20.648	**<0.0001**
**Ferritin, ng/ml**	1.00	1.000 – 1.001	**0.006**	1.00	1.000 – 1.001	**0.029**	1.00	1.001 – 1.002	**<0.0001**			
**Neutrophils**	1.09	1.037 – 1.160	**0.001**				1.65	1.423 – 1.906	**<0.0001**	1.53	1.263 – 1.863	**<0.0001**
**Lymphocytes**	0.88	0.444 – 1.756	0.722				0.32	0.153 – 0.683	**0.003**			
**NLR**	1.05	1.019 – 1.075	**0.001**				1.25	1.161 – 1.348	**<0.0001**			
**AST**	1.00	1.000 – 1.005	0.091				1.02	1.007 – 1.024	**0.001**			
**Symptomatic treatment prior admission**	1.70	0.842 – 3.433	0.139									
**Invasive mechanical ventilation**							5.84	3.188 – 10.705	**<0.0001**	34.2	9.099 – 128.55	**<0.0001**
**Enteral nutrition**							3.09	1.817 – 5.274	**<0.0001**			
**Use of vasopressors**							4.48	2.53 – 7.934	**<0.0001**			
**Days from admission to discharge**							0.97	0.944 – 0.990	**0.005**	0.90	0.856 – 0.947	**<0.0001**
