# Supplementary figures and images for: Time-Dependent Changes of Laboratory Parameters as Independent Predictors of All-Cause Mortality in COVID-19 Patients
Source: Biology (Basel). 2022 Apr 11;11(4):580. doi: 10.3390/biology11040580 (PMC9028239; doi:10.3390/biology11040580)

# Supplementary Figure S1

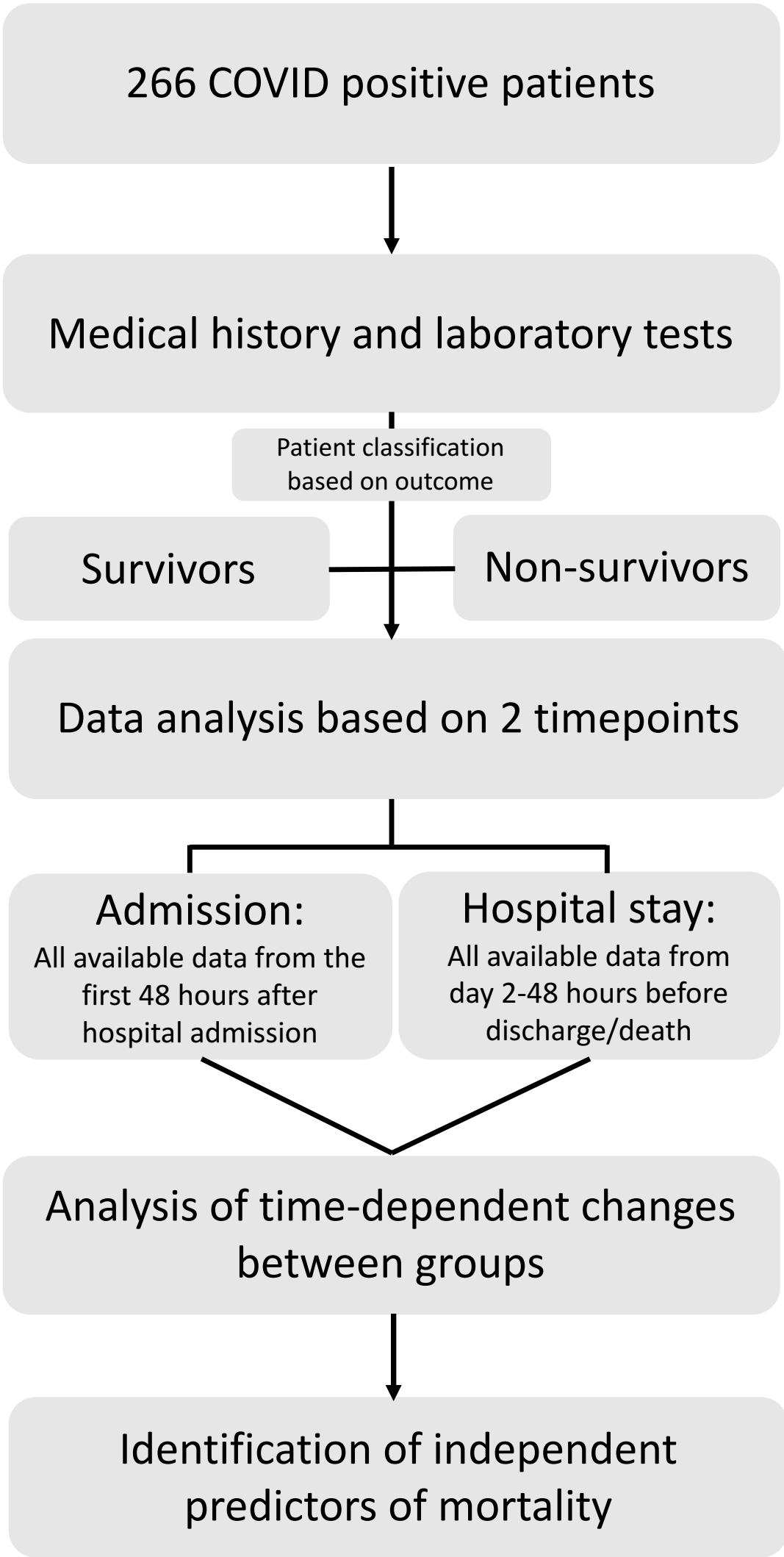

Supplement: Supplementary file 1 [file biology-11-00580-s001.zip › SuppleFigS1-Flow chart.pdf]
